# Supplementary material for: Secure base effect in former shelter dogs and other family dogs: Strangers do not provide security in a problem-solving task
Source: PLoS One. 2021 Dec 22;16(12):e0261790. doi: 10.1371/journal.pone.0261790 (PMC8694447; doi:10.1371/journal.pone.0261790)
Supplement: S1 File — (DOCX) [file pone.0261790.s001.docx]

**
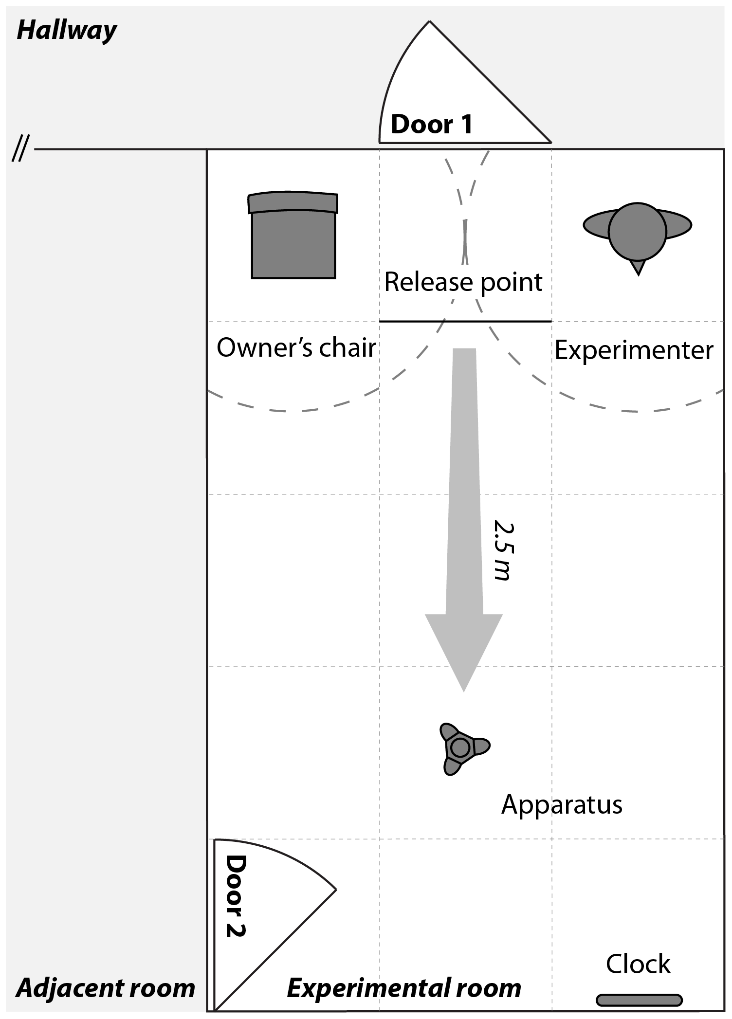
**

**S1 Fig 1. Sketch of the Testing Room.** Adapted from [1].

**
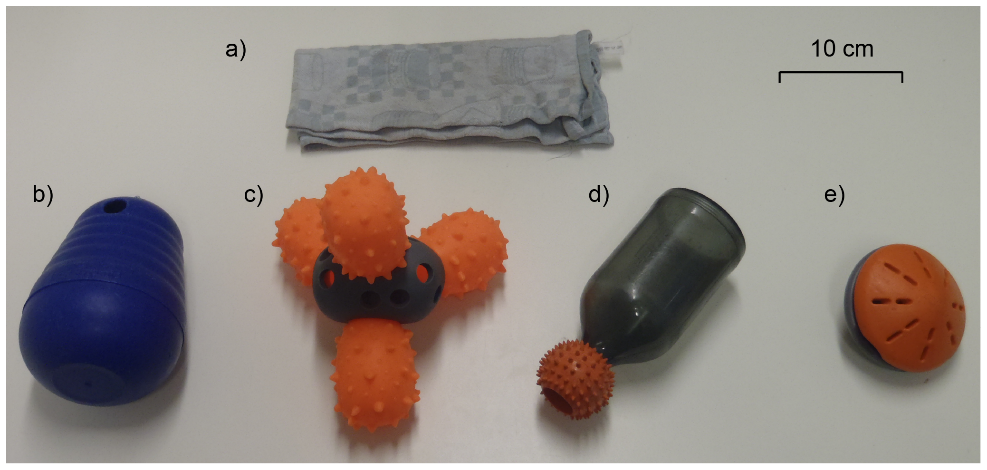
**

**S1 Fig 2. Material used in the test.** a) Kitchen towel which was used in the pre-test, b) c) d) e) four toys which were used in the test (photo from [1])

**S1 Table 1. Complete models results for Experiment 1**

| Dependent variable | Predictor | Estimate; SE | DF | t-value | p-value | Direction of the effect | Post-hoc results (estimate; SE; t-value; p-value) |
| --- | --- | --- | --- | --- | --- | --- | --- |
| Manipulation | **Partner** | **0.76; 0.15** | **1** | **5.03** | **< 0.001** | **O > S** |  |
|  | Behaviour of the partner | 0.02; 0.15 | 1 | 0.16 | 0.88 |  |  |
|  | **Origin of the dog** | **1.03; 0.15** | **1** | **6.67** | **< 0.001** | **Fa > Sh** |  |
|  | Order of condition | - 0.06; 0.07 | 1 | - 0.89 | 0.38 |  |  |
|  | Partner*Behaviour | 0.10; 0.31 | 1 | 0.31 | 0.75 |  |  |
|  | Partner*Origin | 0.30; 0.30 | 1 | 1.01 | 0.32 |  |  |
|  | Behaviour*Origin | 0.20; 0.31 | 1 | 0.65 | 0.51 |  |  |
| Close to Partner | Partner | 0.34; 0.20 | 1 | 1.71 | 0.09 |  |  |
|  | **Behaviour of the partner** | **0.36; 0.14** | **1** | **2.63** | **0.01** | **En > Si** |  |
|  | Origin of the dog | 0.99; 0.19 | 1 | 5.10 | < 0.001 | Sh > Fa |  |
|  | **Order of condition** | **0.14; 0.06** | **1** | **2.32** | **0.02** | **↑** |  |
|  | Partner*Behaviour | 0.44; 0.27 | 1 | 1.62 | 0.11 |  |  |
|  | **Partner*Origin** | **0.94; 0.27** | **1** | **3.47** | **< 0.001** |  | **In Sh: Ow > St (0.59; 0.20; 2.96; 0.004)^a^**  In Fa: Ow = St (0.30; 0.19; 1.52; 0.13) |
|  | Behaviour*Origin | 0.11; 0.27 | 1 | 0.40 | 0.69 |  |  |
| Close to Door | **Partner** | **0.68; 0.16** | **1** | **4.18** | **< 0.001** | **St > Ow** |  |
|  | **Behaviour of the partner** | **0.52; 0.16** | **1** | **3.33** | **0.001** | **Si > En** |  |
|  | **Origin of the dog** | **0.56; 0.16** | **1** | **3.54** | **< 0.001** | **Sh > Fa** |  |
|  | Order of condition | 0.02; 0.07 | 1 | 0.22 | 0.82 |  |  |
|  | Partner*Behaviour | 0.25; 0.32 | 1 | 0.77 | 0.44 |  |  |
|  | Partner*Origin | 0.20; 0.32 | 1 | 0.61 | 0.54 |  |  |
|  | Behaviour*Origin | 0.38; 0.31 | 1 | 1.24 | 0.22 |  |  |
| Exploration | Partner | 0.64; 0.16 | 1 | 4.10 | < 0.001 | St > Ow |  |
|  | **Behaviour of the partner** | **0.37; 0.11** | **1** | **3.43** | **< 0.001** | **En > Si** |  |
|  | Origin of the dog | 0.39; 0.16 | 1 | 2.50 | 0.01 | Sh > Fa |  |
|  | **Order of condition** | **- 0.11; 0.05** | **1** | **- 2.17** | **0.03** | **↓** |  |
|  | Partner*Behaviour | 0.17; 0.22 | 1 | 0.77 | 0.44 |  |  |
|  | **Partner*Origin** | **0.67; 0.22** | **1** | **3.05** | **0.003** |  | In Sh: Ow = St (0.02; 0.16; 0.13; 0.89)  **In Fa: St > Ow (0.68; 0.16; 4.19; < 0.001)^a^** |
|  | Behaviour*Origin | 0.29; 0.22 | 1 | 1.36 | 0.18 |  |  |
| Close to the Experimenter | **Partner** | **0.61; 0.15** | **1** | **3.96** | **< 0.001** | **St > Ow** |  |
|  | Behaviour of the partner | 0.17; 0.15 | 1 | 1.16 | 0.25 |  |  |
|  | **Origin of the dog** | **0.63; 0.15** | **1** | **4.13** | **< 0.001** | **Fa > Sh** |  |
|  | **Order of condition** | **- 0.19; 0.07** | **1** | **- 2.85** | **0.005** | **↓** |  |
|  | Partner*Behaviour | 0.06; 0.30 | 1 | 0.21 | 0.84 |  |  |
|  | Partner*Origin | 0.57; 0.31 | 1 | 1.85 | 0.07 |  |  |
|  | Behaviour*Origin | 0.30; 0.3 | 1 | 0.98 | 0.33 |  |  |

Abbreviations: Owner (Ow), Stranger (St), Shelter (Sh), Family (Fa), Encouraging (En), Silent (Si). In bold are reported the significant results.

^a^ indicates a significant result even after Bonferroni correction for multiple comparisons, in case of post-hoc analysis deriving from analysing constant family and shelter dogs separately (because of a significant interaction).

**S1 Table 2. Complete models results for Experiment 2**

| Dependent variable | Predictor | Estimate; SE | DF | t value | p-value | Direction of the effect |
| --- | --- | --- | --- | --- | --- | --- |
| Manipulation | **Partner (Ow vs. Al)** | **1.08; 0.30** | **1** | **3.64** | **< 0.001** | **Ow > Al** |
|  | Partner (St vs. Al) | 0.22; 0.30 | 1 | 0.73 | 0.47 |  |
|  | **Partner (St vs. Ow)** | **- 0.86; 0.28** | **1** | **- 3.03** | **0.004** | **Ow > St** |
|  | **Origin of the dog** | **1.20; 0.25** | **1** | **4.74** | **< 0.001** | **Fa > Sh** |
|  | Order of condition | 0.01; 0.15 | 1 | 0.06 | 0.96 |  |
|  | Partner (Ow vs. St)*Origin | 0.48; 0.55 | 1 | 0.87 | 0.39 |  |
|  | Partner (Ow vs. Al)*Origin | 0.48; 0.57 | 1 | 0.84 | 0.40 |  |
|  | Partner (Al vs. St)*Origin | 0.00; 0.00 | 0.60 | 0.00 | 1.00 |  |
| Close to Partner | Partner (Ow vs. Al) | 0.30; 0.39 | 1 | 0.77 | 0.45 |  |
|  | Partner (St vs. Al) | 0.39; 0.39 | 1 | 1.02 | 0.31 |  |
|  | Partner (St vs. Ow) | 0.09; 0.38 | 1 | 0.25 | 0.81 |  |
|  | Origin of the dog | 0.94; 0.40 | 1 | 2.34 | 0.02 |  |
|  | Order of condition | 0.13; 0.14 | 1 | 0.93 | 0.36 |  |
|  | Shelter: St vs. Ow | 0.50; 0.44 | 1 | 1.12 | 0.27 |  |
|  | **Shelter: Ow vs. Al** | **1.32; 0.44** | **1** | **2.98** | **0.006^a^** | **Ow > Al** |
|  | Shelter: St vs. Al | 0.82; 0.43 | 1 | 1.92 | 0.07 |  |
|  | Family: St vs. Ow | - 0.90; 0.28 | 1 | - 0.32 | 0.75 |  |
|  | Family: Ow vs. Al | 0.34; 0.30 | 1 | 1.15 | 0.26 |  |
|  | Family: St vs. Al | 0.43; 0.29 | 1 | 1.50 | 0.15 |  |
| Close to Door | **Partner (Ow vs. Al)** | **- 1.05; 0.30** | **1** | **- 3.51** | **< 0.001** | **Al > Ow** |
|  | Partner (St vs. Al) | - 0.40; 0.26 | 1 | - 1.50 | 0.14 |  |
|  | Partner (St vs. Ow) | 0.65; 0.31 | 1 | 2.13 | 0.04 | St > Ow |
|  | **Origin of the dog** | **0.70; 0.24** | **1** | **2.91** | **0.005** | **Sh > Fa** |
|  | Order of condition | 0.01; 0.14 | 1 | 0.05 | 0.96 |  |
|  | Partner (Ow vs. St)*Origin | 0.87; 0.62 | 1 | 1.41 | 0.16 |  |
|  | Partner (Ow vs. Al)*Origin | 0.27; 0.58 | 1 | 0.46 | 0.65 |  |
|  | Partner (Al vs. St)*Origin | - 0.60; 0.52 | 1 | - 1.15 | 0.25 |  |
| Exploration | **Partner (Ow vs. Al)** | **- 0.64; 0.25** | **1** | **- 2.61** | **0.01** | **Al > Ow** |
|  | Partner (St vs. Al) | - 0.13; 0.24 | 1 | - 0.56 | 0.58 |  |
|  | Partner (St vs. Ow) | 0.51; 0.24 | 1 | 2.10 | 0.04 | St > Ow |
|  | Origin of the dog | 0.23; 0.20 | 1 | 1.14 | 0.26 |  |
|  | Order of condition | - 0.05; 0.12 | 1 | - 0.38 | 0.70 |  |
|  | Partner (Ow vs. St)*Origin | - 0.25; 0.48 | 1 | 0.52 | 0.61 |  |
|  | Partner (Ow vs. Al)*Origin | 0.19; 0.48 | 1 | 0.39 | 0.70 |  |
|  | Partner (Al vs. St)*Origin | 0.44; 0.46 | 1 | 0.95 | 0.35 |  |

Abbreviations: Owner (Ow), Stranger (St), Alone (Al), Shelter (Sh), Family (Fa). In bold are reported the significant results.

^a^ indicates a significant result even after Bonferroni correction for multiple comparisons, in case of post-hoc analysis deriving from analysing constant family and shelter dogs separately (because of a significant interaction).

**References**

1. Horn L, Huber L, Range F. The importance of the secure base effect for domestic dogs - evidence from a manipulative problem-solving task. PLoS One. 2013;8: e65296. doi:10.1371/journal.pone.0065296
