# Supplementary material for: Secure base effect in former shelter dogs and other family dogs: Strangers do not provide security in a problem-solving task
Source: PLoS One. 2021 Dec 22;16(12):e0261790. doi: 10.1371/journal.pone.0261790 (PMC8694447; doi:10.1371/journal.pone.0261790)
Supplement: Attachment — Submitted filename: response_to_reviewers.docx [file pone.0261790.s003.docx]

*Rebuttal letter*

*Manuscript PONE-D-21-22971 “Secure base effect in former shelter dogs and other family dogs: strangers do no provide security in a problem-solving task”*

Dear Editor,

Thank you for considering the present manuscript for publication in PLOS ONE. We are pleased to see that the manuscript was positively received and that only few points have been raised by the reviewers that would need to be revised.

Please find below the answers to the points raised by you and by the reviewers. The answers are reported in bold immediately after each point.

Kind regards,

Giulia Cimarelli, PhD (corresponding author)

Journal requirements:

1. Please ensure that your manuscript meets PLOS ONE's style requirements, including those for file naming.

**Done.**

2. Please review your reference list to ensure that it is complete and correct.

**Done.**

Review Comments to the Author

Reviewer #1: The present manuscript reports a study on dog-owner attachment. The goal of the study was twofold: 1) to disentangle between the secure-base effect of owner vs stranger and 2) to investigate how a dog’s history (namely, having lived in the same household since puppyhood vs having lived part of life in a shelter) would influence this secure-base effect. To that end, in two experiments, shelter and family dogs were exposed to a problem-solving task where it was varied whom was present with the dog in the experimental room (owner, stranger, alone), as well as the behavior of the partner (encouraging, silent). It was hypothesized that, if only owners provide a secure-base effect to their dogs, dogs would spend more time trying to solve the problem when the owner was present than when the stranger was present or the dog was alone. Secondly, encouragement was expected to facilitate object manipulation (i.e., dogs would persevere more), at least in the presence of the owner. Finally, differences in behavior were expected to emerge as a function of dogs’ past history. Three major results were found:
1) Owners, but not strangers, provided dogs with a secure base effect – dogs manipulated the object longer in the presence of the owner than in the presence of the stranger or when alone;
2) This effect was independent of the owner’s behavior (silent vs encouraging)
3) The above mentioned effects did not depend on dogs’ past history. However, there were some differences in the behavior of the two groups, with shelter dogs being overall warier in the novel environment and in relation to the stranger (exhibiting more behavioral signs correlated with anxiety).
Authors concluded that both dogs that have lived since puppyhood in the same household and dogs that have been re-homed can develop a secure attachment to their owners, but that having spent some time in a shelter can hinder these dogs’ ability to cope with new environments and people.

The study was conducted with rigor, the methods are overall well-presented and the results support the conclusions. However, some more effort has to be put in the Introduction section, on the presentation of the state of the art as well as on the derivation and presentation of hypothesis. Specifically, because the study has two research questions that are answered by crossing/combining the results of the two conducted experiments, the authors need to further work on presenting the state of the art that informs their two questions and to better explain how they relate to each other, as well as to clarify the derivation of their hypothesis. I think this can be achieved mainly with some re-writing and re-organization of the Introduction section. I detail my comments in what follows.

Introduction
First, I think the Introduction needs to be shortened. I feel five pages is a lot concerning the size of the current paper but, more importantly, shortening will allow the authors to be more straightforward and direct when presenting the theoretical background and the gap that the current piece of research aims at filling. Some examples/suggestions are:

**We shortened and restructured the Introduction, making it more concise than before.**

- Condense what is presented in paragraphs between lines 114 and 142. I think such a detailed presentation of Horn’s study is not necessary.

**We drastically reduced this part and condensed the previous three paragraphs into one (lines 115-131).**

- Paragraphs between line 143 and 171: Can the authors condense and present the ideas in a more straightforward way? The level of methodological detail given here is, in my opinion, not necessary.

**We reduced the explanation of the methods to the minimum. The paragraph now extends from line 132 to line 139.**

- As it is currently written, it almost seems that the Introduction will end on line 113. However, after that, the authors still discuss the issue on disentangling between owner and stranger secure-base effects, and afterwards they go back to shelter vs family dogs again. I would advise a reorganization in a way where the reader is not jumping from one topic to the other.

**We restructured the Introduction according to the following: general intro about the secure base effect 🡪 the secure base effect in dogs and the problem with previous studies: owner-stranger differentiation not always clear, especially for those dogs changing caregiver throughout their lifetime (e.g. guide dogs) 🡪 presentation of the phenomenon that former shelter dogs might differ from family dogs acquired since puppyhood in owner-stranger differentiation because of their life experiences 🡪 short presentation of the methods and aim for each experiment 🡪 hypotheses and predictions for the overall owner-stranger differentiation and for the former shelter vs. constant family dogs potential differences.**

An additional suggestion is that the hypotheses are only presented in the final paragraph of the Introduction. Personally, I do not think the authors need to go into so much detail regarding hypotheses in this section (currently there are several hypotheses presented across the Introduction). I would rather opt by presenting more general hypotheses (and the specific goals of each Experiment, which is not done in the current version of the manuscript) and maybe latter (for example, in the Methods section) present all the more “specific” hypotheses in detail. If the authors decide they want to present all the detailed hypotheses, I would strongly advise presenting them using bullet points, such that it is easier for the reader to follow and consult when needed.

**We have moved all hypotheses and predictions to the end of the introduction (lines 140-162) and wrote them in a more straightforward manner. We think that listing each prediction using bullet points is not necessary anymore.**

Another comment I have to the Introduction concerns the ideas presented in lines 59-64:
“It is important to realise, however, that such an owner-stranger differentiation depends not only on the strength of the dog’s bond to its owners but also on the ease with which a dog accepts strangers as social partners. Stranger acceptance varies greatly across individual dogs and does so independent of the dogs’ bonds to their owners [3,10–12]. Many studies, however, do not make an effort to disentangle these two factors…” -- I wonder whether the authors aimed at disentangling this owner bond vs stranger acceptance effects in the present study. If they did, it is not currently clear how. If they did not, then maybe this specific issue can be dropped out of the Introduction.

**I the present study we could directly investigate stranger’s acceptance/social support in the experimental situation thanks to the comparison with the alone condition (Experiment 2). That is, we did not only compare the behaviour of dogs in the presence of the owner and in the presence of the stranger, but compared both conditions to the alone one. Moreover, the comparison between the two groups (former shelter dogs and constant family dogs) helped us disentangling the two options. In order to make this points clearer, we revised the aims, hypotheses and predictions paragraphs at lines 137-139, 145-147 and 154-162.**

Line 162-163: Please state earlier that an experimenter was always present in the room in Experiment 1, such that it is clear from early on that this was the case.

**Done. It is not at lines 134-135.**

Methods
Please state early on in this section (in the Experimental Design and General Procedure) when the experimenter was or was not present in the room (in Experiment 1, Experiment 2 and pre-tests).

**We stated this early on at lines 135-139, but also again 199-201 in the methods section.**

Line 245 - Who was holding the dog while “she placed it on the marked spot in the experimental room”?

**The owner. Added at line 218.**

Lines 248-249 - Why this difference between conditions? Why didn’t the owner and the stranger entered together as well in the “owner condition”?

**There was a reason only in the stranger condition for sending the owner into the room together with the stranger: had the owners stayed in the corridor from where the dog entered the room, the dogs would have likely hesitated to enter the room, thereby creating a difference between conditions (e.g. dogs showing less interest in the toys not because the owner was not in the room but because they stick to the door that separates them from the owner). There was no reason, however, to send the stranger into the room in the owner condition. On the contrary, this, unnecessarily, would have made the strangers more familiar for the dogs – an effect we wanted to avoid in the current experiment where two different persons played the role of the stranger in the two stranger conditions.**

Line 259: “At the end of the trial” – The information on what determined the end of a trial should be presented already here (currently it is only presented later, on line 270)

**Added at lines 228-229.**

Line 260 – Please add to where the experimenter “left the testing room together with the dog” (hallway?)

**Added at line 231-232.**

The info on lines 271-280 should come in line 268 right after “first in presence and then in absence of their owner”.

**We moved the information regarding each trial up. Now at lines 241-250.**

Lines 406-408: I think the paper benefits if this info is presented earlier (see comment above).

**The presence of the experimenter in the room in Experiment 1 but not in Experiment 2 is already mentioned at lines 135-139, 199-201, 374, 385-386, hence multiple times and earlier than this point.**

Results
I think there are too many graphs/tables in this section. Please think reducing the number of figures – maybe create two big figures (one for Experiment 1 and one for Experiment 2) with several panels. Moreover, I would suggest moving the tables to supplementary material.

**We combined figures as suggested. Now there are only two figures for Experiment 1 and one figure for Experiment 2. Moreover, we moved the Tables to the Supporting Information and uploaded them separately from the main manuscript in the S1 File.**

Discussion
My only comment to this section is that I missed seeing the present results compared/contrasted with those from Previde and Valsecchi (2007) [6, 17], the only other published study comparing attachment in shelter vs ‘family’ dogs.

**Thank you for this comment. We added that indeed our in line with that previous study. We added this information at lines 531-540.**
