# Supplementary material for: Secure base effect in former shelter dogs and other family dogs: Strangers do not provide security in a problem-solving task
Source: PLoS One. 2021 Dec 22;16(12):e0261790. doi: 10.1371/journal.pone.0261790 (PMC8694447; doi:10.1371/journal.pone.0261790)
Supplement: Attachment — Submitted filename: response_to_reviewers_02122021.docx [file pone.0261790.s004.docx]

**Response to reviewers - PLOS ONE manuscript [PONE-D-21-22971R1]**

Dear Editor,

Please find below the answers to each of your and the reviewers’ points. Our answers are reported in bold below each point.

Thank you very much for your work and valuable contribution to improve the present manuscript.

Kind regards,

Giulia Cimarelli, PhD

Additional Editor Comments:
Line 1 "do no**t**" (the letter t is missing)

**Corrected.**

Line 31 "affects" (the letter s is missing)

**Corrected.**

Line 132 Replace "[26]'s" with the author name followed by [26]

**Corrected.**

Line 168 What are the GSP guidelines? I'm not familiar with them, and googling gave me a reference to a trade term.

**Good Scientific Practice. We corrected it.**

Line 249 Start a new paragraph at "The dog passed the pre-test"

**Done.**

Line 315 The term "partner" has as far as I can see not been defined previously. I could deduce that it was the person present with the dog in the test situation, but I suggest you define the term here or in the methods section, where you introduce the social conditions around the set-up

**Added at line 273 and 289.**

Line 486 Replace "would have had an effect" with "had an effect"

**Corrected.**

Line 489 Replace "calls attention that" with "highlight that" or "calls attention to that"

**Corrected.**

Line 593 "teacher**s**" (the letter s is missing)

**Corrected.**

Comments to the Author

6. Review Comments to the Author

Reviewer #1: I would like to thank the authors for the changes and clarifications provided and for the detailed responses to my comments. I think the manuscript has improved substantially. I would just like some minor issues to be addressed before I can say the paper is ready for being published. I detail my suggestions and questions in what follows.

Terms
The terms “constant family dogs” and “former shelter dogs” make the text too verbose sometimes. I understand why the authors choose these terms but, personally, for a matter of easiness of reading, I would use “family dogs” and “shelter dogs”. This is just a suggestion, and I am ok with the authors deciding to stick to the current terms.

**Thank you for your suggestion, we thought about it but we don’t want to confuse the reader: by simply mentioning shelter and family dogs, the risk is that the reader will interpret shelter dogs as still being host in the a shelter, while all dogs in our sample are indeed family dogs, but one group has a past in a shelter. We would like to stick with the terminology used so far, even if a but verbose.**

Aims of study
I would suggest that the authors keep the presentation of the aims of the study more consistent throughout the paper. I do not mind, and actually think it is helpful for the reader, if a very similar wording (or even the same) is used.
In the end of the Introduction (lines 163-165), the authors state two aims:

- “to compare the secure base effect of owners to the social support strangers may provide dogs with”
- “to investigate how having lived in a shelter may influence stranger acceptance and bonding with the owner.”

Latter, in the beginning of the Discussion (lines 452-456), despite the authors stating the study had two main aims, I personally see three. Namely:
- “to investigate whether strangers could serve as a secure base for pet dogs”
- to investigate “to what extent they would do so less than owners would”
- “to examine whether the secure base effect provided by owners (and potentially by strangers) would differ between dogs with a past in the shelter and dogs who have always lived with the same owner.”

**Thank you. We modified the end of the Introduction (Lines 165-168) to more clearly state the 3 aims of the study and be more in line with the rest of the manuscript, as the reviewer suggested: “Overall, the present study allows us to test whether strangers could serve as a secure base for pet dogs and to compare the secure base effect of strangers to the one provided by owners. Moreover, the present study aims at investigating how having lived in a shelter may influence stranger acceptance and bonding with the owner.” Moreover, we changed that the study aims are three and not two as previously stated at line 464.**

Line 122: I would suggested reorganizing to something like:
However, in the first study using this paradigm [26], some methodological limitations did not allow to fully compare to what extent owners and strangers could serve as a secure base for dogs. Specifically, while there was a condition in which the owner would be encouraging the dog, no corresponding condition existed for the stranger. Although results showed that dogs spent more time manipulating the object when the owner was encouraging them as compared to when the stranger was silent, and no differences were found between the silent owner and the silent stranger conditions, the possibility is still open that also an encouraging unfamiliar human could facilitate dogs’ manipulation, when compared to the owners.

**Thank you! Indeed the structure of the paragraph is simpler and clearer. We modified as the reviewer suggested.**

Line 140-147: Here I would suggest stating which the hypothesis are for each Experiment. -- lines 140-144 refer to results to be obtained in Experiment 1 and lines 145-157 refer to results to be obtained in Experiment 2.

**Yes, we added in brackets to what experiment each prediction is referred to (Lines 146 and 149).**

Line 148-162: This second part is still a bit confusing. As far as I understand, the authors are presenting three hypothesis, namely:
1. dogs might be very flexible and could be able to build new bonds easily, even when adults, resulting in no differences between constant family and former shelter dogs.
2. dogs’ re-homing past might have an effect on their social development and as such…
2.1. “Having lived in a shelter negatively affects the future development of a secure attachment bond or
2.1. “Having lived in a shelter increases the likelihood of accepting strangers as social partners
3. former shelter dogs might form a secure attachment bond with their owners comparable to the one formed by other family dogs, but be generally more anxious than constant family dogs

…but the way it is currently written does not leave it clear.

**Thank you. We corrected that indeed the hypotheses are 3 (Line151) and we added list item letters for the in-sentence list. This should make the structuring clearer (paragraph at lines 150-164).**

Still in Line 154: “In the first case, former shelter dogs would manipulate and explore less in the presence of the owner than other family dogs.” – what about Owner-Stranger differentiation? One would expect a less strong O-S differentiation, no?

**Yes, we added “and a less strong owner-stranger differentiation”.**

Line 200 - I suggest introducing here the concept “coordinating experimenter” and using it every time from here on.

**Thanks for you suggestion, we added it and used it throughout the text.**

Line 202 - Why not referring here to the towel as well? Doesn’t this section refer to the Experimental Setup for both the pre-test and the test? Moreover, in the Materials section, the authors present the materials for both pre-test and test.

**Yes, we added it at line 206.**

Line 218: Consider writing “held by the owner on a leash” instead of “held by the owner thanks to the leash”.

**Corrected.**

Moreover, was the dog watching the coordinating experimenter from the hallway or inside the room?

**From the hallway, we added it at line 223.**

Please clarify throughout the section Experimental Design and General Procedure when were the doors opened or closed. For example, in line 221 “The experimenter and the dog waited outside the testing room in the hallway” – was Door 1 opened or closed?

**Closed. We added details regarding whether the door was open or closed throughout the whole section.**

Line 280 - When the authors write “Similarly to the study by [26], “Manipulation” was coded whenever the dog was touching the toy with its muzzle or paw” it seems that it was just for this behavior and not the following ones. Please clarify if the remaining coded behaviors were also coded “similarly to [26]”.

**All behaviours were coded following [26]. We specified this at line 291 and we used semicolons instead of colons to list the different variables.**

Line 506-509: “It is difficult to tell whether former shelter dogs’ exploring to a similar extent in presence of the owner and of the stranger was due to lower stress when the owners were absent or more support when they were present.”
This idea is hard to follow. Maybe the problem is the fact that it is not clear whether these comparisons (“lower stress when the owners were absent” and “more support when they were present”) refers to a comparison between shelter dogs and family dogs or if it refers to a within-group comparison (i.e., owner-stranger comparison for shelter dogs). Namely, when the authors say that these dogs could be showing lower stress in the absence of the owner, does this mean lower as compared to what family dogs would show, or lower as compared to the other condition (owner present)? It is also confusing because, as far as I understand, exploration in this paragraph seems to be interpreted both as a sign of distress and a sign of secure-base effect. In this same example, when the authors are trying to interpret the absence of differences in exploration in shelter dogs, it seems that the first hypothesis (lower stress when the owners were absent) is based on distress-related exploration and the second hypothesis (more support when they were present) is based on exploration related to a secure-base effect provided by the owner.
I truly feel this section needs clarification.

**Only within the former shelter group. And yes, exploration could be either a sign of distress or of a secure base effect. We modified the sentence to make this clearer “If exploration was a sign of distress also in the case of former shelter dogs, then a similar duration of exploration in the presence of the owner and of the stranger might be either due to low stress when the owner is absent (stranger condition) or to received support when the owner is present (owner condition).” We additionally simplified the rest of the paragraph (lines 515-527).**

Typos:
Abstract line 31: remove “s” in “affects”.

**Corrected.**

Line 350: I believe the authors mean “Experiment 1”

**Corrected.**
Line 453: I suggest removing the “would” at the end of the sentence

**Corrected.**
